# Negative association between right bundle branch block and inducibility of ventricular tachyarrhythmias in Chagas disease

**DOI:** 10.1590/S1679-45082014CE3126

**Published:** 2014

**Authors:** Henrique Horta Veloso

I read with interest the article by Cedraz et al.^(1)^ describing their findings on the electrophysiological study of 115 chagasic patients. The authors reported that the presence of right bundle branch block (RBBB) was associated with less induction of sustained ventricular tachycardia or ventricular fibrillation (VT/VF) in a univariate analysis. In 56 patients with RBBB, VT/VF was induced in 35 (37.5%), *versus* 21 of 59 cases (57.6%) without this disturbance (p=0.03). This difference resulted in an *odds ratio* of 0.44 (confidence interval 95%; 0.21-0.93). Unfortunately, the authors did not discuss this finding.

Some studies have shown the inducibility of VT/VF as a predictor of mortality in Chagas disease.^(2-4)^ The presence of RBBB was already demonstrated as an independent predictor of mortality in a multivariate analysis.^(4,5)^

Therefore, the negative association between RBBB and inducibility of VT/VF in chagasic patients is a unique observation that should not be disregarded and needs further investigation.

Henrique Horta Veloso Cardioteam, Hospital do Rio, Rio de Janeiro, RJ, Brazil.

## AUTHOR’S REPLY

We are grateful for the interest and comments of our colleague on our study. We agree that the association between right bundle branch block and lower level of induction of ventricular arrhythmias in Chagas patients is unprecedented and interesting. However, this is only an initial observation in a reduced number of patients. Future investigations, including studies properly designed for this purpose and with adequate population samples are needed for any definitive cause and effect relation between right bundle branch block and ventricular arrhythmia induction to be established.

The present study has a purely descriptive design and is not intended to determine causal relations. The association found between a lower level of ventricular arrhythmia induction and right bundle branch block was only observed in the univariate analysis, and was not confirmed in the multivariate analysis, which suggests that the association is not causal. For this reason, and in order to avoid an erroneous interpretation on the part of readers, we chose to not discuss the finding, although it is possible that the methodological aspects explained above might be incorporated into the discussion of the manuscript, pointing out the unique aspect of this finding as well as its limitations.

Luciana Armaganijan e Dalmo Moreira

Instituto Dante Pazzanese de Cardiologia, São Paulo, Brazil.

## References

[1] Cedraz SS, Silva PC, Minowa RK, Aragão JF, Silva DV, Morillo C (2013). Electrophysiological characteristics of Chagas disease. einstein.

[2] Silva RM, Távora MZ, Gondim FA, Metha N, Hara VM, Paola AA (2000). Predictive value of clinical and electrophysiological variables in patients with chronic chagasic cardiomyopathy and nonsustained ventricular tachycardia. Arq Bras Cardiol.

[3] Leite LR, Fenelon G, Simoes A, Silva GG, Friedman PA, Paola AA (2003). Clinical usefulness of electrophysiologic testing in patients with ventricular tachycardia and chronic chagasic cardiomyopathy treated with amiodarone or sotalol. J Cardiovasc Electrophysiol.

[4] Rassi A, Rassi A, Rassi SG (2007). Predictors of mortality in chronic Chagas disease: a systematic review of observational studies. Circulation.

[5] Rodriguez-Salas LA, Klein E, Acquatella H, Catalioti F, Davalos VV, Gomez-Mancebo JR (1998). Echocardiographic and clinical predictors of mortality in chronic Chagas’ disease. Echocardiography.

