# Supplementary material for: Negative association between right bundle branch block and inducibility of ventricular tachyarrhythmias in Chagas disease
Source: Einstein (Sao Paulo). 2014 Jul-Sep;12(3):389. doi: 10.1590/S1679-45082014CE3126 (PMC4872959; doi:10.1590/S1679-45082014CE3126)
Supplement: Translation

# Translation: Associação negativa entre bloqueio do ramo direito e indutibilidade de taquiarritmias ventriculares na cardiopatia chagásica

**Authors:** Henrique Horta Veloso

Li com interesse o estudo de Cedraz et al.,^(1)^ que descreveram os achados do estudo eletrofisiológico de 115 chagásicos. Os autores demonstraram que a presença do bloqueio do ramo direito (BRD) esteve relacionada a menor indução de taquicardia ventricular sustentada ou fibrilação ventricular (TV/FV) pela análise univariada. Nos 56 pacientes com BRD, TV/FV foi induzida em 35 (37,5%) contra 21 de 59 casos (57,6%) sem essa alteração (p=0,03). Essa diferença resultou em uma *odds ratio* de 0,44 (intervalo de confiança de 95% de 0,21-0,93). Infelizmente, não houve discussão quanto a esse achado.

Alguns estudos demonstraram a indutibilidade de TV/FV como preditor de mortalidade na cardiopatia chagásica.^(2-4)^ Quanto ao BRD, sua presença já foi demonstrada como preditora independente de mortalidade em análise multivariada.^(4,5)^

Portanto, a associação negativa entre BRD e indutibilidade de TV/FV em chagásicos é um achado original, que não deve ser desprezado, e suscita interesse para investigações mais aprofundadas.

Henrique Horta Veloso Cardioteam − Hospital do Rio, Rio de Janeiro, RJ, Brasil.

## RESPOSTA DO AUTOR

Agradecemos o interesse e os comentários do colega sobre nosso estudo. Concordamos que a associação entre bloqueio de ramo direito e menor indução de arritmias ventriculares em pacientes chagásicos encontrada é inédita e interessante, entretanto, essa é apenas uma observação inicial e em número reduzido de pacientes. Futuras investigações, incluindo estudos propriamente desenhados para esse fim e com amostra populacional adequada, são necessárias para que se possa estabelecer qualquer relação de causa e efeito definitiva entre bloqueio de ramo direito e indução de arritmias ventriculares.

A presente pesquisa possui um desenho puramente descritivo e não almeja determinar relações de causalidade. A associação encontrada entre menor indução de arritmias ventriculares e bloqueio de ramo direito foi observada apenas na análise univariada e não confirmada na análise multivariada, sugerindo que tal associação não é causal. Por esse motivo e para se evitar uma interpretação errônea por parte dos leitores, optamos por não discutir tal achado, embora, talvez, os aspectos metodológicos explicados acima pudessem ter sido incorporados à discussão do manuscrito, salientando, o aspecto inédito deste achado, bem como suas limitações.

Luciana Armaganijan e Dalmo Moreira

Instituto Dante Pazzanese de Cardiologia, São Paulo, Brasil.
